# Cancer Cell Gene Expression Modulated from Plasma Membrane Integrin αvβ3 by Thyroid Hormone and Nanoparticulate Tetrac

**DOI:** 10.3389/fendo.2014.00240

**Published:** 2015-01-12

**Authors:** Paul J. Davis, Gennadi V. Glinsky, Hung-Yun Lin, John T. Leith, Aleck Hercbergs, Heng-Yuan Tang, Osnat Ashur-Fabian, Sandra Incerpi, Shaker A. Mousa

**Affiliations:** ^1^Department of Medicine, Albany Medical College, Albany, NY, USA; ^2^Pharmaceutical Research Institute, Albany College of Pharmacy and Health Sciences, Rensselaer, NY, USA; ^3^Stanford University, Palo Alto, CA, USA; ^4^Taipei Medical University, Taipei, Taiwan; ^5^Rhode Island Nuclear Science Center, Narragansett, RI, USA; ^6^Cleveland Clinic, Cleveland, OH, USA; ^7^Hematology Institute and Blood Bank, Meir Medical Center, Kfar-Saba, Israel; ^8^Department of Medicine, Sackler Faculty of Medicine, Tel Aviv University, Tel Aviv, Israel; ^9^Department of Sciences, University Roma Tre, Rome, Italy

**Keywords:** integrin, thyroid hormone, tetraiodothyroacetic acid, nanoparticle, gene transcription

## Abstract

Integrin αvβ3 is generously expressed by cancer cells and rapidly dividing endothelial cells. The principal ligands of the integrin are extracellular matrix proteins, but we have described a cell surface small molecule receptor on αvβ3 that specifically binds thyroid hormone and thyroid hormone analogs. From this receptor, thyroid hormone (l-thyroxine, T_4_; 3,5,3′-triiodo-l-thyronine, T_3_) and tetraiodothyroacetic acid (tetrac) regulate expression of specific genes by a mechanism that is initiated non-genomically. At the integrin, T_4_ and T_3_ at physiological concentrations are pro-angiogenic by multiple mechanisms that include gene expression, and T_4_ supports tumor cell proliferation. Tetrac blocks the transcriptional activities directed by T_4_ and T_3_ at αvβ3, but, independently of T_4_ and T_3_, tetrac modulates transcription of cancer cell genes that are important to cell survival pathways, control of the cell cycle, angiogenesis, apoptosis, cell export of chemotherapeutic agents, and repair of double-strand DNA breaks. We have covalently bound tetrac to a 200 nm biodegradable nanoparticle that prohibits cell entry of tetrac and limits its action to the hormone receptor on the extracellular domain of plasma membrane αvβ3. This reformulation has greater potency than unmodified tetrac at the integrin and affects a broader range of cancer-relevant genes. In addition to these actions on intra-cellular kinase-mediated regulation of gene expression, hormone analogs at αvβ3 have additional effects on intra-cellular protein-trafficking (cytosol compartment to nucleus), nucleoprotein phosphorylation, and generation of nuclear coactivator complexes that are relevant to traditional genomic actions of T_3_. Thus, previously unrecognized cell surface-initiated actions of thyroid hormone and tetrac formulations at αvβ3 offer opportunities to regulate angiogenesis and multiple aspects of cancer cell behavior.

## Introduction

Integrins are heterodimeric structural proteins of the plasma membrane and are principally involved in cell–cell relationships in tissues and cell–extracellular matrix (ECM) protein interactions (1). The extracellular domain of an integrin binds specific ECM proteins and outside-in transmission of the occurrence of liganding results in the generation of specific signals by the intra-cellular domain of the integrin. These signals, usually involving various kinases, may result in cellular changes in actin (2, 3) and cell motility (4), modulate endocytosis (5), and affect transcription of specific genes (6–8).

Amply expressed by and activated in cancer cells, integrin αvβ3 interacts with ECM proteins, but has recently been shown to have a panel of specific receptors for non-protein, small molecule ligands (9). Among these are sites for the binding of thyroid hormone (10, 11), dihydrotestosterone (12), and resveratrol (13). The thyroid hormone receptor (TR) on αvβ3 has been well-studied (11, 14). What is now apparent is that this receptor has more complex and coherent effects on cancer-relevant gene expression than had been apparent in analyses of the impact of large molecule (protein) interactions with the integrin. The multiple genes whose expression is modulated from the extracellular domain of αvβ3 by thyroid hormone or its derivative, tetraiodothyroacetic acid (tetrac), relate to angiogenesis, cancer cell proliferation, metastasis, and cancer cell defense pathways (15). The latter include genes relevant to anti-apoptosis, anti-angiogenesis, chemoresistance (MDR1), and repair of double-strand DNA breaks induced by radiation. Within the cell, unmodified tetrac mimics certain actions of thyroid hormone. At the extracellular domain of αvβ3, in contrast, tetrac blocks binding of l-thyroxine (T_4_) and 3,5,3′-triiodo-l-thyronine (T_3_) – that is, it is a thyroid hormone antagonist. Covalent bonding of tetrac to a nanoparticle prevents cell entry of tetrac and, compared to unmodified tetrac, broadens the spectrum of defensive cancer cell genes whose expression can be desirably regulated from the integrin. This expanded panel includes pro-apoptotic genes and epidermal growth factor receptor (EGFR) gene (see subsequent sections). In addition, the potency of nanoparticulate tetrac as a thyroid hormone antagonist at αvβ3 is greater than that of unmodified tetrac. Thus, without entering the cancer or endothelial cell, thyroid hormone analogs non-genomically initiate important actions on tumor cell and blood vessel cell gene expression. In this review, we survey αvβ3-mediated effects of thyroid hormone and analogs on gene expression in human cancer cells, analyzed by RT-PCR. We also point out that, from its receptor on the integrin, thyroid hormone has adjunctive effects on nuclear receptors for thyroid hormone and for estrogen, regulating the state of phosphorylation or acetylation of such receptors and controlling the formation of complexes within the nucleus of coactivators and receptors.

## Early Evidence That Thyroid Hormone Could Modulate Gene Expression from the Cell Exterior: Protooncogene Expression; Angiogenesis

Prior to the discovery of the plasma membrane receptor for thyroid hormone and hormone analogs on integrin αvβ3, agarose-T_4_ had been shown to regulate protooncogene expression (16, 17). Agarose-T_4_ is a prototypic nanoparticulate formulation of l-thyroxine in which T_4_ is covalently bound to a linear polysaccharide polymer; the product is excluded from the cell interior. The thyroid hormone effect on gene expression in these studies was mitogen-activated protein kinase (MAPK)-dependent and was reproduced in cells that lacked the nuclear TR.

Studied in the chick chorioallantoic membrane (CAM) model and also prior to recognition of the hormone receptor on αvβ3, T_4_ at physiological free concentrations and T_3_ at concentrations that were supraphysiologic were shown to increase vascularity threefold in 72 h (18). The degree of activity was comparable to that of fibroblast growth factor 2 (FGF2; bFGF). Agarose-T_4_ also reproduced the pro-angiogenic effect of thyroid hormone. The effects of unmodified thyroid hormone and of agarose-T_4_ on angiogenesis were found to be inhibited by tetrac, the hormone analog subsequently shown to block the iodothyronine receptor site on the cell surface. Pharmacologic inhibitors of MAPK (ERK1/2) and of protein kinase C also eliminated thyroid hormone-induced angiogenesis. RT-PCR studies revealed that the hormone-induced transcription of *FGF2* within 6 h, and measurement of FGF2 protein in the medium showed increased release of the angiogenic factor. Thus, the promotion of vascular sprouting (19) and new vessel formation by thyroid hormone was attributable to initiation at the plasma membrane of a non-genomic effect culminating in expression of a specific vascular growth factor gene, manufacture of the gene product and release of the latter protein into the medium.

The cell surface receptor for thyroid hormone and tetrac was shortly thereafter defined on the extracellular domain of integrin αvβ3 and functionally described in the context of angiogenesis (10). Other thyroid hormone agonist analogs, such as GC-1 (20) and diiodothyropropionic acid (DITPA) (21) were also shown to be pro-angiogenic, and tetrac blocked the activity of these analogs at the integrin. However, the anti-angiogenic properties of tetrac expressed at the integrin extend beyond the blockade of binding of T_4_ and T_3_ to αvβ3. As discussed in the next section, tetrac or its reformulation as a nanoparticulate may affect expression of blood vessel-relevant genes beyond *FGF2* independently of T_4_ and T_3_. Tetrac and Nanotetrac may also disrupt crosstalk between αvβ3 and adjacent receptors for other vascular growth factors, such as vascular endothelial growth factor (VEGF) and FGF2 (22), and platelet-derived growth factor (PDGF) (Shaker A. Mousa, unpublished observations). However, these effects on crosstalk are unrelated to gene transcription.

## Tetrac, Nanotetrac, and Gene and microRNA Expression That is Relevant to Angiogenesis

As indicated above, unmodified tetrac is taken up by cells and expresses low-grade T_4_-like activity and may be converted to triiodothyroacetic acid (triac), which is also thyromimetic (23, 24). To limit the action of tetrac exclusively to integrin αvβ3, we covalently bonded tetrac to a nanoparticle of sufficient size (~200 nm) to preclude cell uptake of the complex (25), thus mimicking agarose-T_4_. The polymer we used was biodegradable poly(lactic-co-glycolic acid), in contrast to the physiologically inert agarose. The nanoparticulate formulation involved a stable ether bond of the outer ring hydroxyl group of tetrac to a 6-carbon linker and amide-bonding of the latter to PLGA (25). The amide bond was imbedded in the nanoparticle and thus not readily accessible to circulating or tissue peptidases. The resulting Nanotetrac indeed was restricted to the extracellular space and preserved the previously known actions of tetrac, but it was also found to have desirable additional biologic activities not previously obtained with tetrac.

Microarray studies of two human cancer cell lines showed that tetrac and Nanotetrac downregulated expression of *VEGFA* (26), the gene product of which is a principal inducer of the porous blood vessels associated with cancers (27). These effects are initiated at plasma membrane αvβ3. Tetrac and Nanotetrac also increased transcription of thrombospondin 1 (*THBS1*, *TSP1*). TSP1 protein is an endogenous suppressor of angiogenesis and is invariably suppressed in cancer cells. Nanotetrac also decreased expression of *EGFR*, the gene product of which mediates actions of EGF on angiogenesis. Tetrac lacked this action. Nanotetrac, but not tetrac, downregulates expression of *NF*κ*B* via the integrin and NFκB de-activation is an anti-angiogenic target (28, 29). Finally, thyroid hormone may regulate transcription of the monomeric α*v* gene (30), but it is not known whether this action is initiated at the αvβ3 protein or requires the nuclear TR.

In recent studies of microRNA (miR), we have shown that Nanotetrac increases cellular abundance of miR-15A in breast cancer cells by 10-fold (31) and decreases miR-21 by 50%. miR-21 is pro-angiogenic in certain tumor cells (32) and miR-15A decreases angiogenesis by a VEGF-dependent mechanism (33).

Transcriptional mechanisms involved in the anti-angiogenic activity of Nanotetrac at αvβ3 are summarized in Table 1.

**Table 1:** **Transcriptional mechanisms by which Nanotetrac/tetrac is anti-angiogenic**.

Angiogenesis-relevant target	Action	References
bFGF transcription	↓	(18)
VEGFA transcription	↓	(11, 26)
EGFR transcription	↓	(34)
TSP1 (THBS1) transcription	↑	(26, 34)
miR-21 transcription	↓	(31)
miR-15A transcription	↑	(31)
Cellular bFGF abundance	↓	(18)
Cellular Ang-2 abundance	↓	(22)
Cellular MMP-9 abundance	↓	(35)
Pro-angiogenic activity of thyroid hormone	↓	(36)

*Measurements of gene transcription were made in breast cancer (34) and medullary thyroid carcinoma cells (26). Protein abundance decreases are presumed to reflect decreased expression of specific genes. The pro-angiogenic activity of thyroid hormone involves non-transcriptional mechanisms as well as actions on specific gene expression shown in this table. Mechanisms that appear to be non-transcriptional are crosstalk between integrin αvβ3 and adjacent vascular growth factor receptors on the cell surface, cell release mechanisms for newly synthesized growth factors and regulation of endothelial cell motility (11, 18, 36)*.

## Thyroid Hormone Supports Cancer Cell Proliferation and is Anti-Apoptotic; Tetrac–Nanotetrac Transcriptionally Inhibits Cancer Cell Proliferation, is Pro-Apoptotic and Disrupts Cell Defense Pathway Gene Expression

A number of laboratories have described the stimulatory effect of thyroid hormone on tumor cells (37–43) and clinical studies have defined thyroid hormone dependence of cancers, in that spontaneous or medically induced hypothyroidism has improved outcomes (44–49). Trophic actions of thyroid hormone on tumor cells were presumed to require a TR isoform and to be genomic in mechanism – that is, to require physical interaction of a TR protein and T_3_ – until recognition of the existence in 2005 (10) of the cell surface receptor for thyroid hormone and tetrac on αvβ3, described above (11, 50). Existence of this receptor offered a discrete, non-genomic mechanism for initiation of tumor cell proliferation. TRβ may be involved in certain cancer cell proliferative responses to thyroid hormone (51, 52), but work by Cheng and co-workers indicates that TRβ is a tumor suppressor that, when mutated in the thyroid gland, may be oncogenic (53).

The demonstration that T_4_ – including the agarose-T_4_ formulation – was a proliferative factor for certain human tumor (breast, thyroid cancer) (42, 54) and animal cells (C6, F98, GL261 glioma cell lines) (43) was accompanied by evidence that unmodified tetrac inhibited the T_4_ effect. We had shown that unmodified tetrac blocked non-genomic actions of thyroid hormone on plasma membrane functions (11, 14, 55). The proliferative effect was MAPK-dependent. Interestingly, in human breast cancer (MCF-7) cells, tetrac-inhibitable enhancement of proliferation by thyroid hormone involved Ser-118 phosphorylation of nuclear estrogen receptor-α (ERα); this pathway is identical to that by which estrogen stimulates MCF-7 cell proliferation (54).

In a model of resveratrol-induced apoptosis that involved MAPK phosphorylation of p53 at Ser-15, we showed that T_4_ was anti-apoptotic. The hormone prevented the p53 phosphorylation step in several tumor cell lines (42, 56). Tetrac blocked this anti-apoptotic activity of T_4_. Additional evidence of the anti-apoptotic activity of T_4_ included inhibition of nucleosome liberation by resveratrol, as well as cellular accumulation of the pro-apoptotic BcLx_s_ protein (56, 57). The hormone did not, however, affect cell accumulation of survival protein BcLx_l_. The action of thyroid hormone on nucleosome liberation and BcLx_s_ in tumor cells was prevented by tetrac (58).

Subsequent microarray studies conducted with Nanotetrac in human breast cancer (MDA-MB-231) cells revealed a coherent pro-apoptosis pattern of gene expression. That is, transcription of the X-linked inhibitor of apoptosis (*XIAP*) gene was downregulated and transcription of a set of pro-apoptotic genes – *CASP2*, *CAP8AP2*, *DFFA*, and *BCL2L14* – was stimulated (11, 34).

We would also note that Nanotetrac downregulates expression of 8 of 9 cyclin genes and 1 cyclin-dependent kinase gene (34) and more than 20 oncogenes.

## Tetrac–Nanotetrac Actions on Expression of Genes Relevant to Tumor Invasiveness

Catenins are proteins involved in cell–cell adhesion. β-catenin also has transcriptional functions in the nucleus. Mutation and overexpression of the β-catenin gene occurs in a variety of cancers, including colorectal carcinoma, breast, and ovarian cancer (59, 60). Nanotetrac increases transcription of the *CBY1* gene (34), the gene product of which is an inhibitor of nuclear functions of β-catenins. This is a desirable action of Nanotetrac at αvβ3 in cancer cells. The action would be deleterious in non-cancer cells, but the latter when not undergoing cell division express little αvβ3. Like β-catenins, integrin αvβ3 participates in cellular adhesion complexes.

Nanotetrac also affects α-catenins, downregulating expression of the *CTNNA1* and *CTNNA2* genes. Mutation of *CTNNA2* is associated with tumor invasiveness and thus inhibition of transcription of the gene is desirable, as is downregulation of the non-mutated gene in cancers. The non-mutated gene product of *CTNNA1* can function as a tumor invasion suppressor (61), but mutation is associated with gastrointestinal tract and other cancers (62).

As mentioned above, *MMP-9* expression is induced by thyroid hormone (35). The observations were recently made in myeloma cells and were inhibited by tetrac, thus implicating αvβ3 in this action of T_4_. This action of the hormone may contribute to local extension of myeloma in bone and, if documented to be present in solid tumor cells, may presage metastasis. *MMP-2* transcription may also be subject to control by thyroid hormone (63, 64). Several mechanisms may be involved in the hormonal action on *MMP-2*, and it is not yet known whether this effect of the hormone is initiated at integrin αvβ3. The importance of this is that an intact metalloproteinase axis interferes with cell–cell interaction, resulting in tissue destabilization and support of cancer cell invasiveness and metastasis (65).

## Overview of Anti-Cancer Properties of Nanotetrac, Acting as a Single Modality

The anti-cancer actions of Nanotetrac are broadly based in terms of mechanisms, despite initiation at a single target receptor on integrin αvβ3, and in this regard resemble the pluralistic anti-angiogenic actions of the drug. As noted above, the coherence of the effects of the agent on expression of differentially regulated cancer cell genes is remarkable. It is possible that there are effects of Nanotetrac at αvβ3 that may involve integrity of the actin cytoskeleton in cancer cells, and that the drug may influence interactions of the integrin with ECM proteins that may disorient tumor cell movement or interfere with defensive responses (see Conjunctive Radiation and Tetrac/Nanotetrac Treatment of Cancer Cells: Radiosensitization below). However, these possibilities have not yet been examined.

Nanotetrac promotes apoptosis, antagonizes anti-apoptotic (survival) defenses, disrupts control of the cell cycle, and interferes with function of the frequently mutated catenins (11, 26, 34). As noted above in the review of angiogenesis, thyroid hormone and tetrac or its Nanotetrac formulation affect matrix metalloproteinase gene expression. We would also note that thyroid hormone (T_4_) has protein-trafficking action on integrin αvβ3, directing internalization of the membrane protein – without the hormone ligand – and nuclear uptake of the αv monomer, but not of β3. In the nuclear compartment, αv is a coactivator (66) involved in transcription of a number of important cancer-relevant genes (see below, Adjunctive Modifications of Nuclear Hormone Receptors that Originate at the Hormone Receptor on αvβ3; Nuclear Uptake of αv Monomer).

Some of these actions of Nanotetrac/tetrac are summarized in Table 2.

**Table 2:** **Mechanisms of selected cancer chemotherapeutic actions of Nanotetrac**.

Action	Example	References
Chemosensitization	Decreased efflux of doxorubicin, P-gp effect; increased effectiveness of other chemotherapeutic agents	(67)
Radiosensitization	Disordered repair of radiation-induced double-strand DNA breaks; prevention of radiation-induced activation of integrin ávâ3	(68–70)
Disabling of cell survival pathway gene expression	Decreased expression of anti-apoptotic *XIAP*, *MCL1*; enhanced expression of pro-apoptotic *CASP2*, *BCL2L14*, *TP53*, *PIG3*, *BAD*; disruption of catenin pathways via increased expression of *CBY1*, decreased expression of *CTNNA1*, *CTNNA2*; decreased expression of pro-oncogenic *miR-21*, increased expression of pro-apoptotic *miR-21*; decreased expression of matrix metalloproteinase genes, e.g., *MMP-9*; decreased expression of stress-defense genes, e.g., *HIF-1α*, decreased expression of multiple *Ras* oncogenes	(11, 26, 34, 35, 71)
Cell cycle	Downregulation of multiple cyclin, cyclin-dependent protein kinase genes	(11, 34)
Disordering of growth factor pathways	Suppression of *EGFR* gene expression, disabled function of EGFR	(11, 34)
	See Table 1 for other activities vs. other vascular growth factors, receptors	

## Chemosensitization by Tetrac of Cancer Cells Resistant to Other Cancer Chemotherapeutic Agents

P-glycoprotein (P-gp; MDR1; ABCB1) is a plasma membrane efflux pump whose ligands include a number of cancer chemotherapeutic agents (72). The pump is a principal component of cancer cell chemoresistance. Thyroid hormone causes transcription of *MDR1* (73–75) and increases function of the P-gp protein (75). Thus, ambient thyroid hormone may be viewed as a support mechanism for chemoresistance (76). It is not known what the molecular basis is for regulation by iodothyronines of P-gp function or *MDR1* gene expression, i.e., microarray studies have not been conducted to establish whether the induction of *MDR1* gene expression is dependent upon the hormone receptor on integrin αvβ3. However, tetrac increases the intra-cellular retention time of doxorubicin by doxorubicin-resistant breast cancer cells (67), an effect attributed to action of tetrac–Nanotetrac on pump function of P-gp or on gene expression (76).

## Conjunctive Radiation and Tetrac/Nanotetrac Treatment of Cancer Cells: Radiosensitization

Hercbergs and co-workers have defined the potentiation of radiation exposure by tetrac in animal glioma (C6) cells (68) and human glioblastoma (U87MG) cells (69), and Nanotetrac in human prostate cancer (PC3, LNCaP) cells (70). *In vitro* studies revealed that at a 4 Gy x-radiation dose 1 h after exposure to tetrac, there is a 60% reduction in cell survival, compared to control (68). The mechanism of action of tetrac and Nanotetrac is interference with cancer cell repair of double-strand DNA breaks (neutral comet assay/mean tail moment) (69). What components of the DNA break repair process – and, specifically, transcription of what specific genes – are affected by tetrac/Nanotetrac is not yet known.

## Adjunctive Modifications of Nuclear Hormone Receptors That Originate at the Hormone Receptor on αvβ3; Nuclear Uptake of αv Monomer

The above discussion relates to regulation of transcription of specific cancer cell genes by thyroid hormone analogs that act at the cell surface via integrin αvβ3. Relevant additionally to the end result of modulation of transcription of specific genes from integrin αvβ3 is the adjunctive input from the integrin to the state of nuclear TRs. We have recently reviewed this subject (77). In brief, trafficking of cytoplasmic TRβ1 to the cell nucleus is directed by T_4_ at the integrin via MAPK, and the importing by the nucleus of TRα1 is promoted by T_3_ via activation of phosphatidylinositol 3-kinase (78). Two discrete binding domains exist at the TR site on αvβ3: the S1 site binds T_3_ exclusively and S2 binds both T_4_ and T_3_. Tetrac–Nanotetrac interferes with hormone binding at both domains. In the case of TRβ1 trafficking, translocation of the receptor into the nucleus occurs as a complex with activated MAPK; specific phosphorylation of the receptor (activation) is a consequence (79, 80). An example of specific gene transcription that occurs as a result of this trafficking/phosphorylation is expression of hypoxia-inducible factor-1α (*HIF1α*) in response to T_3_ at the S1 site (78). The complex process of stimulating cancer cell or endothelial cell proliferation occurs via the S2 domain.

Integrin αvβ3 may be internalized by cells as a result of the protein’s liganding of T_4_ (13). The αv monomer is imported by the nucleus as a result of this process and has been shown to be a coactivator protein that binds to the promoter region of a number of genes, including *ERα*, *HIF-1α*, cyclooxygenase-2 (*COX-2*), and *TR*β1. ERα protein is important to breast, ovarian, and certain lung cancers. We have implicated nuclear COX-2 protein in the pharmacologic induction of apoptosis (58). HIF-1α protein is a cell survival factor that triggers angiogenesis and cellular conversion to anaerobic metabolism (81). The αv monomer does not import thyroid hormone and the β3 monomer is not taken up by the nucleus. This remarkable process was an unexpected consequence of studies of small molecule actions at the integrin and offers a novel mechanism for regulation of gene expression from the cell surface and integrin.

## Conclusion

Integrin αvβ3 controls a variety of intra-cellular and transcellular functions. It is a transmembrane structural protein that is differentially expressed/activated in tumor cells and dividing blood vessel cells. The definition of the specific thyroid hormone-tetrac receptor site on αvβ3 (10, 11, 14) enabled recognition of the existence of control from a single locus of expression of differentially regulated, angiogenesis-relevant genes as well as modulation of function of adjacent vascular growth factor receptors. Nanotetrac is a prototypic anti-angiogenic and anti-cancer agent focused on a single, specific small molecule receptor site on the extracellular domain of αvβ3. From this site, Nanotetrac blocks actions of VEGF, FGF2, and PDGF at their plasma membrane receptors, inhibits expression of *VEGFA* and *EGFR*, stimulates transcription of *TSP1*, decreases endothelial cell abundance of Ang-2 without affecting Ang-1, selectively regulates miRNAs that control angiogenesis and decreases endothelial cell motility (Table 1).

From the standpoint of anti-cancer activity, Nanotetrac desirably disrupts gene expression critical to cell cycling in αvβ3-bearing tumor cells and dividing endothelial cells and interferes with a substantial group of cell survival pathways so that apoptosis is advanced, and defensive anti-apoptosis pathways are disordered (Table 2). Nanotetrac reverses chemoresistance and confers radiosensitivity. This novel and extensive spectrum of actions makes Nanotetrac an attractive anti-angiogenic and anti-cancer agent for further development. The agent has been shown to be an effective anti-proliferative, pro-apoptotic agent in a variety of human cancer cell lines (25, 31, 40–43, 71), to be effective against human cancer xenografts (26, 57, 70, 82–84) and to include important downregulation of tumor-associated angiogenesis (22, 26, 36, 57, 82, 84).

In the absence of an agent such as Nanotetrac with anti-thyroid hormone activity at integrin αvβ3, a reduction in circulating thyroid hormone, notably T_4_, that is either spontaneous or medically induced appears to be effective in slowing clinical growth of certain solid tumors. These include breast (45), glioblastoma multiforme (44), head-and-neck cancers (47), and renal cell carcinoma (46). We can postulate that such reductions in systemic levels of T_4_ largely affect tumors via the examples of gene expression reviewed above. Several of the current authors have recently confirmed clinically that systematic reduction in circulating T_4_ (euthyroid hypothyroxinemia) may arrest growth of certain cancers (85).

## Conflict of Interest Statement

Paul J. Davis and Shaker A. Mousa are co-inventors of Nanotetrac. The issued and pending patents for Nanotetrac are held by NanoPharmaceuticals LLC, a company from which the inventors receive no reimbursement. The other co-authors declare that the research was conducted in the absence of any commercial or financial relationships that could be construed as a potential conflict of interest.
