# Corrigendum: “Cancer Cell Gene Expression Modulated from Plasma Membrane Integrin αvβ3 by Thyroid Hormone and Nanoparticulate Tetrac”

**DOI:** 10.3389/fendo.2015.00098

**Published:** 2015-06-09

**Authors:** Paul J. Davis, Gennadi V. Glinsky, Hung-Yun Lin, John T. Leith, Aleck Hercbergs, Heng-Yuan Tang, Osnat Ashur-Fabian, Sandra Incerpi, Shaker A. Mousa

**Affiliations:** ^1^Department of Medicine, Albany Medical College, Albany, NY, USA; ^2^Pharmaceutical Research Institute, Albany College of Pharmacy and Health Sciences, Rensselaer, NY, USA; ^3^Stanford University, Palo Alto, CA, USA; ^4^Taipei Medical University, Taipei, Taiwan; ^5^Rhode Island Nuclear Science Center, Narragansett, RI, USA; ^6^Cleveland Clinic, Cleveland, OH, USA; ^7^Hematology Institute and Blood Bank, Meir Medical Center, Kfar-Saba, Israel; ^8^Department of Medicine, Sackler Faculty of Medicine, Tel Aviv University, Tel Aviv, Israel; ^9^Department of Sciences, University Roma Tre, Rome, Italy

**Keywords:** integrin, thyroid hormone, tetraiodothyroacetic acid (tetrac), nano particle, gene expression, corrigendum

In Table 2, the section designated “Disabling of cell survival pathway gene expression,” line 5 incorrectly states “…pro-apoptotic miR-21”; the correct statement is “…pro-apoptotic miR-15A.” The issue is correctly discussed in the body of the published text, p. 2, right column, paragraph 4.

## Conflict of Interest Statement

The authors declare that the research was conducted in the absence of any commercial or financial relationships that could be construed as a potential conflict of interest.

